# Treating Bell’s Palsy With Osteopathic Manipulative Medicine: A Case Report

**DOI:** 10.7759/cureus.11092

**Published:** 2020-10-22

**Authors:** Mikhail Volokitin, Asad Sheikh, Sapan Patel, Susan Milani, Mary Banihashem

**Affiliations:** 1 Osteopathic Medicine, Touro College of Osteopathic Medicine, New York, USA; 2 Osteopathic Medicine, New York Institute of Technology College of Osteopathic Medicine, Old Westbury, USA; 3 Osteopathic Medicine, Touro College of Osteopathic Medicine, Middletown, USA

**Keywords:** bell’s palsy, facial nerve (cn vii) paralysis, osteopathic manipulative treatment, osteopathic manipulative medicine

## Abstract

Bell's palsy (BP) occurs when the facial nerve (CN VII) is swollen, inflamed, or compressed, resulting in facial weakness or paralysis; etiology is unknown. BP patients often succumb to a decreased quality of life due to the inability to make facial expressions, increased sensitivity to auditory stimuli, and dysregulation in tear and saliva production. Despite conventional examination and therapy options, the syndrome is majorly regarded as idiopathic and left unresolved for many patients. In this case of a patient with BP, treatment with osteopathic manipulative treatment (OMT) which focused on restoring a normal structure-function relationship resolved the patient’s symptoms. The osteopathic manipulative procedures utilized findings from an osteopathic structural exam and addressed those somatic dysfunctions with OMT. The authors report that the patient's symptoms improved after the application of OMT and without the use of pharmaceuticals. The results of the case study suggest that treating BP with OMT can rapidly improve symptoms and can be used without or concurrently with other treatment modalities, if applicable. Patient’s consent for this case report was obtained in written and verbal form.

## Introduction

Bell's palsy (BP), or idiopathic facial paralysis, occurs when the facial nerve, or CN VII, is swollen, inflamed, or compressed, resulting in facial weakness or paralysis. While the exact etiology is unknown, often a viral etiology is suspected but unproven [1]. BP afflicts approximately 40,000 Americans each year or approximately 20-30 in 100,000 persons [1-3]. Patients with BP often succumb to unilateral facial paralysis, decreased tearing, hyperacusis, and/or loss of taste perception. Beyond the direct clinical features of BP, many patients are also met with psychological distress from the debilitation of BP.

## Case presentation

A 32-year-old African-American female presented to an Osteopathic Manipulative Medical practice with right-sided facial weakness, an inability to close her right eye, dryness in her right eye, and tears running down her right cheek for several weeks. The patient reported no pain but complained of difficulties with activities of daily living (ADL): inability to chew correctly, keep food in the mouth, drooling on the right side, inability to close the right eye in the shower, and at night. The patient also experienced increased sensitivity to sound and a decreased sensitivity to taste. The patient’s symptoms had started three months prior to seeking treatment from an osteopathic physician. The patient admits to feeling anxious and having sleeping difficulties from increased stress attributed to the recent death of her father, employment situation as a ride-share driver and telemarketer, and lack of improvement of her condition. Prior to her initial visit, the patient reported receiving 12 acupuncture sessions, using lubricating eye drops in her right eye, taking oral ibuprofen 400 mg twice daily and oral prednisone 60 mg daily, sliding scale for five days. The patient not only reported a lack of improvement, but also the progressive worsening of her symptoms before deciding to visit an osteopathic physician.

On physical examination, bilateral enlarged salivary glands (parotid, sublingual, and submandibular glands) were noted with the right side larger than the left. The right preauricular, postauricular, and cervical lymph nodes were found moderately enlarged and tender. Other findings included palpation of a horseshoe kidney, bilaterally enlarged inguinal lymph nodes, and tenderness in the left and right lower quadrants of the abdomen with diminished gastrointestinal motility with auscultation.

 An osteopathic structural exam (OSE) was conducted on the initial visit to reveal pain and tenderness on right facial muscles, right suboccipital muscles, right cervical flexor and extensor muscles, right sternocleidomastoid, right trapezius, right levator scapulae, and right scalene muscles. The cephalad motion of the sternum and ribs 2-5 were also restricted with a decreased range of motion (ROM) at the left sternochondral junction upon exhalation. The right hemidiaphragm was found to be restricted. Decreased ROM was noted in the left sacroiliac (SI) joint.

 The complete osteopathic somatic dysfunction (SD) diagnosis includes cranial SD (left side-bending/rotation strain of the sphenoid, right occipital condyle anterior compression, right occipitomastoid suture compression, internally rotated right temporal bone), cervical SD (OA F RlSr; C3 F RrSr), thorax SD (sternum cephalad motion restriction, ribs 2-5 inhalation SD, right hemidiaphragm restriction), sacral SD (right on right torsion) and lastly, pelvic SD (left posterior innominate).

A diagnosis of BP, or idiopathic facial nerve paralysis, was made based on the patient’s clinical symptomatology of sudden unilateral facial and neck paralysis, excessive tearing, mouth dryness, hyperacusis, and a loss of taste. The anatomic location of the facial nerve lesion was determined to be distal to the geniculate ganglion due to the presence of tearing. Axonal damage was suspected due to the lack of symptom improvement for over three months. While electromyography (EMG) was considered to confirm or rule out axonal damage, an EMG referral was postponed until treatment trial and further observation.

On the initial visit, based on the patient's history of the absence of clinical improvement for more than three months, facial nerve atrophy was considered, and the prognosis was guarded. Upon completion of treatment on the third visit, the patient's prognosis was assessed as favorable for complete recovery. The diagnosed somatic dysfunctions were treated with occipital condyle release, occipitomastoid suture decompression, and temporal rocking, among others, to resolve structural restriction impinging CN VII. 

**Figure 1:**
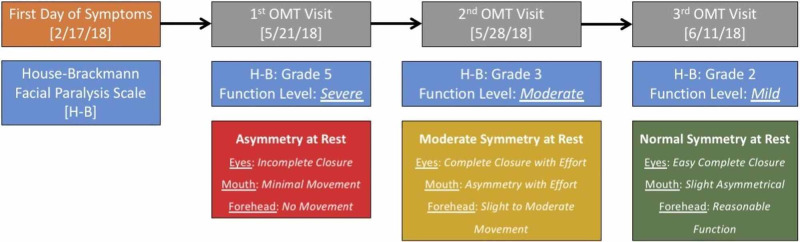
Patient visits with HB facial paralysis scale. The HB scale was used to assess the facial nerve dysfunction at each visit. While initially unable to smile on the right side of her face or close her right eye (Grade V), the patient regained a significant amount of motor function and was able to better fulfil ADL, with her smile more symmetrical and the patient no longer using an eye patch/taping while showering (Grade III). Following her third visit (11th June 2018) the patient mentioned that she felt an “85% improvement” in her symptoms (Grade II). HB, House-Brackmann; ADL, activities of daily living; OMT, osteopathic manipulative treatment

One week after the patient received the first treatment (21st May 2018), the patient’s symptoms visibly improved, and EMG was not warranted. After the second visit (28th May 2018), her symptoms had improved noticeably. The House-Brackmann (H-B) scale was used to evaluate the facial nerve function. While initially unable to smile on the right side of her face or close her right eye (Grade V), the patient regained a significant amount of motor function and was able to better fulfil ADL, with her smile more symmetrical and the patient was no longer using an eye patch/taping while showering (Grade III). Following her third visit (11th June 2018) the patient mentioned that she felt an “85% improvement” in her symptoms (Grade II). This can be seen in Figure 1. It was noted that neither symptom was fully resolved in comparison to her unaffected side. The H-B scale was used to assess the facial nerve dysfunction at each visit [4].

## Discussion

Medical societies recommend treating BP by taking advanced sequential approaches based on the presence or lack of improvement in patient symptoms. If there is no spontaneous recovery with observation, the American Academy of Neurology (AAN) and the American Academy of Otolaryngology-Head and Neck Surgery Foundation (AAO-HNSF) indicate the use of pharmacologic therapy such as corticosteroids and antiviral therapy within 72 hours from the onset of symptoms [5-6].

Based on the OMM philosophy, biomechanical, neurological, and psychosocial models were applied while evaluating the patient. With clear symptoms indicating a neurological dysfunction, further etiological causes were considered. Negative history of infection ruled out viral etiology. The treatment plan was focused on OMT addressing any biomechanical and psychological factors. The diagnosed somatic dysfunctions were treated with occipital condyle release, occipitomastoid suture decompression, and temporal rocking, among others, to resolve structural restriction impinging CN VII. The presence of tearing gave the reason to believe the anatomic compression was distal to the geniculate ganglion. Lymph circulation is important when resolving edematous and inflammatory processes. Lymphatics are a low-pressure system that relies primarily on interstitial fluid pressure and the pumping effect of extrinsic muscle contractions, meaning that external pressure on lymphatic vessels can alter the body’s ability to resolve drainage and flow problems. The glymphatic system in the central nervous system (CNS) is responsible for waste clearance and metabolic solute distribution [7]. Dysfunction and deterioration of the glymphatic system can lead to increased aggregation of solutes and metabolic waste and thus further amplify an injury [8]. Lymphatic techniques, such as thoracic inlet, sternum, diaphragmatic release, and pedal pumps were utilized to improve restricted lymphatic circulation and impaired lymphatic drainage causing the patient’s symptoms and impeding her recovery. Secondary somatic dysfunction, such as increased cervical muscle tone, which can affect the proper lymphatic drainage from the head and face, was addressed with myofascial soft tissue techniques, counterstain, myofascial release, and muscle energy (ME) techniques. Ribs SD were addressed with rib raising and ME, aiming at decreasing the sympathetic output. Sacral SDs were addressed with balanced ligamentous tension (BLT), pelvic with ME. By treating this patient with OMT, we were able to restore most functions in her facial nerve. Lastly, breathing and meditation exercises were taught to the patient to address her stress-related symptoms.

In BP, paralysis of the orbicularis oculi muscle happens, the patient cannot blink, and the cornea dries out. The dryness and irritation of the cornea, signaled by CN V-1 (part of the afferent limb of the blink reflex), stimulate the superior salivatory nucleus, which in turn, sends secretomotor signals via the greater petrosal nerve, CN VII, to the lacrimal gland to produce and release an increased volume of tears. The presence of tearing is helpful in the anatomical localization of the lesion. The parasympathetic fibers branch from the facial nerve at the geniculate ganglion and become the greater petrosal nerve. As a result, if a lesion is proximal to the geniculate ganglion, all facial nerve modalities, including tearing, will be lost. As the tearing was present in this patient, we conclude that her right facial nerve was affected distal to the geniculate ganglion [9].

Typically, in BP there is no axonal damage. However, sometimes axonal damage does occur and there is electromyographic evidence of muscle degeneration in the face. In this case, recovery is prolonged because it is dependent on the regeneration of the nerve, which may take years. After axonal damage of the facial nerve, there may be regeneration with the risk of misdirection of the regenerating fibers. When encountering a patient with a suspected diagnosis of BP, osteopathic practitioners should focus on evaluating and addressing the following structural abnormalities along the facial nerve pathway and the adjacent areas; dura mater (posterior cranial fossa, tentorium cerebelli, C1-C2), temporal bone - petrous pyramid, internal acoustic meatus, occipito-mastoid suture, occipital condyles, sphenobasilar symphysis, temporomandibular joint [10].

## Conclusions

The application of osteopathic manipulative treatment (OMT) was focused on addressing cranial, cervical, sacral somatic dysfunction responsible for the symptoms of the patient’s unilateral facial nerve paralysis. No medications were prescribed. OMT was focused on restoring normal structural relationships and improving lymphatic circulation, which revolved around relieving the compression forces on the facial nerve pathway and releasing the tension in tissues along the lymphatic drainage pathways.

Indirect sphenoid dysfunction correction, occipital condyle release, and occipitomastoid suture decompression were the primary techniques used to address diagnosed cranial somatic dysfunction, while counterstain and ME were used to address secondary somatic dysfunction in the rest of the body. The results suggest that treating BP with OMT can rapidly improve symptoms and may be used without or concurrently with allopathic treatments, if applicable. Larger studies may be needed to provide statistically significant scientific evidence for the use of OMT for BP patients.
